#  Queima de biomassa da cana-de-açúcar e hospitalizações de crianças e idosos por agravos respiratórios em Pernambuco, Brasil 

**DOI:** 10.1590/0102-311XPT238422

**Published:** 2023-11-13

**Authors:** Renata Cordeiro Domingues, Aline do Monte Gurgel, Romário Correia dos Santos, João Antonio dos Santos Pereira, Virgínia Carmem Rocha Bezerra, Wayner Vieira de Souza, Mariana Olívia Santana dos Santos, Idê Gomes Dantas Gurgel

**Affiliations:** 1 Instituto Aggeu Magalhães, Fundação Oswaldo Cruz, Recife, Brasil.; 2 Instituto de Saúde Coletiva, Universidade Federal da Bahia, Salvador, Brasil.; 3 Programa de Pós-graduação em Desenvolvimento e Meio Ambiente, Universidade Federal de Pernambuco, Recife, Brasil.

**Keywords:** Poluentes Atmosféricos, Biomassa, Cana-de-Açúcar, Doenças Respiratórias, Air Pollutants, Biomass, Sugarcane, Respiratory Tract Diseases, Contaminantes Atmosféricos, Biomasa, Caña de Azúcar, Enfermedades Respiratorias

## Abstract

Este estudo buscou analisar a relação entre as hospitalizações por agravos respiratórios e a queima regular da cana-de-açúcar em Pernambuco, Brasil. Trata-se de um estudo ecológico de série temporal correspondente ao período de 2008 a 2018. Foram comparadas as taxas de hospitalizações por agravos respiratórios em crianças menores de 5 anos e em idosos maiores de 60 anos em municípios produtores e não produtores de cana-de-açúcar, por meio da análise estatística não paramétrica de Mann-Whitney. Conjuntamente, foi observada a distribuição mensal das ocorrências de focos de calor nos municípios casos e controles e aplicada a correlação de Pearson para analisar a associação entre ambas as variáveis. Foi verificado que, para ambos os grupos etários, as taxas de hospitalizações são maiores nos municípios produtores de cana-de-açúcar, com diferença estatística significativa p < 0,005. A taxa de internação hospitalar em idosos é 28% mais elevada nos municípios casos, sendo ainda maior em crianças menores de 5 anos, cuja razão das medianas é 40%. No entanto, foi identificado que o comportamento sazonal das hospitalizações por agravos respiratórios diverge do observado na distribuição mensal dos focos de calor, não havendo correlação estatística significativa. Esses achados sugerem possível associação com a exposição crônica aos particulados emitidos pela queima de biomassa, comprometendo a saúde de grupos vulneráveis, e endossam a necessidade de substituição das queimadas no monocultivo da cana-de-açúcar, bem como a estruturação de políticas públicas de proteção à saúde humana e ambiental.

## Abstract (en)

This study aimed to analyze the relationship between hospitalizations for respiratory problems and the regular burning of sugarcane in Pernambuco State, Brazil. This is an ecological time series study corresponding to the period from 2008 to 2018. The rates of hospitalizations for respiratory diseases in children aged under 5 years and in adults older than 60 years in sugarcane-producing and non-producing municipalities were compared using nonparametric Mann-Whitney statistical analysis. Together, we observed the monthly distribution of the hot spots occurrences in the case and control municipalities and applied Pearson’s correlation to analyze the association between both variables. For both age groups, hospitalization rates are higher in sugarcane-producing municipalities, with a statistically significant difference p < 0.005. The rate of hospitalization in older adults is 28% higher in the case municipalities, and is even higher in children aged under 5 years whose ratio of the medians is 40%. However, the seasonal behavior of hospitalizations for respiratory diseases differs from that observed in the monthly distribution of hot spots, without statistically significant correlation. These findings suggest a possible association with chronic exposure to particulates emitted by biomass burning, compromising the health of vulnerable groups, and endorse the need to replace fires in the monoculture of sugarcane and to structure public policies to protect human and environmental health.

## Resumen

Este estudio buscó analizar la relación entre las hospitalizaciones por enfermedades respiratorias y la quema regular de caña de azúcar en Pernambuco, Brasil. Se trata de un estudio ecológico de serie temporal correspondiente al período entre 2008 y 2018. Las tasas de hospitalizaciones por enfermedades respiratorias en niños menores de 5 años y en ancianos mayores de 60 años en municipios productores de caña de azúcar y en los municipios no productores de azúcar se compararon mediante el análisis estadístico no paramétrico de Mann-Whitney. Se observó en conjunto la distribución mensual de las ocurrencias de puntos calientes en los casos y controles de los municipios, y se aplicó la correlación de Pearson para analizar la asociación entre ambas variables. Se encontró que, para ambos grupos de edad, las tasas de hospitalización fueron más altas en los municipios productores de caña de azúcar, con una diferencia estadísticamente significativa p < 0,005. La tasa de hospitalización de los ancianos fue un 28% mayor en los municipios casos, y aún mayor que la de los niños menores de 5 años cuya relación de las medianas fue del 40%. Sin embargo, se identificó que el comportamiento estacional de las hospitalizaciones por enfermedades respiratorias difiere de lo observado en la distribución mensual de puntos calientes, sin correlación estadística significativa. Estos hallazgos evidencian una posible asociación con la exposición crónica a partículas emitidas por la quema de biomasa, lo que afecta la salud de los grupos vulnerables, además apuntan a la necesidad de implementar medidas contra los incendios en el monocultivo de la caña de azúcar y políticas públicas para proteger la salud humana y el medioambiente.

## Introdução

A agropecuária brasileira caracteriza-se por grandes monocultivos e pela elevada dependência do uso de insumos químicos ^1^. A produção do país é voltada fundamentalmente para a produção de *commodities* agrícolas, com destaque para a soja, cana-de-açúcar e milho ^2^, o que tem posicionado o país entre aqueles com as maiores produções agrícolas do mundo ^3^. A cana-de-açúcar destaca-se na produção extrativista desde os tempos coloniais da *sacarocracia*^4^, com forte expressão no mercado internacional sucroenergético.

A queima da palha da cana-de-açúcar é prática dos tempos coloniais, que antecede a colheita e é realizada sobretudo para viabilizar a produtividade por meio do corte manual em regiões de relevos mais acidentados ^5^. Ela pode desencadear incêndios de grandes proporções, principalmente em épocas de grande estiagem, e intensifica problemas de saúde na população ^6^. De acordo com a Organização Mundial da Saúde OMS ^7^, a exposição a poluentes atmosféricos segue sendo associada a uma gama variada de efeitos danosos à saúde humana, sobretudo agravos respiratórios e cardiovasculares, incluindo aumento do risco de morte. No Brasil, estudos têm associado a prevalência e a internação hospitalar por doenças respiratórias agudas em populações expostas à fumaça proveniente desta prática ^8^^,^^9^^,^^10^^,^^11^.

Os problemas de saúde podem estar associados à exposição ao material particulado ^6^, assim como às substâncias químicas emitidas na atmosfera durante a queima, como as dioxinas, reconhecidamente carcinogênicas para humanos ^12^. Sabe-se que, quanto maior a proximidade com as áreas de queimadas, maior a exposição humana às emissões atmosféricas e aos seus efeitos danosos à saúde. Entretanto, a direção e a intensidade das correntes de ar também exercem influência sobre a dispersão dos poluentes atmosféricos, ampliando as áreas afetadas pela pluma oriunda do fogo, como acontece quando os ventos predominantes se dirigem para áreas urbanas ou áreas densamente povoadas, sujeitando um número maior de pessoas aos efeitos nocivos dos contaminantes aéreos ^13^^,^^14^.

Os grupos mais suscetíveis aos efeitos deletérios da poluição atmosférica são crianças, sobretudo abaixo de 5 anos de idade, idosos e indivíduos com histórico de doenças respiratórias e cardiovasculares ^6^^,^^8^^,^^15^^,^^16^^,^^17^. As infecções respiratórias agudas, asma e bronquite, estão relacionadas aos altos níveis de poluição aérea e são causas comuns de morbimortalidade entre os grupos de maior vulnerabilidade.

Buhler et al. ^18^ evidenciaram, em estudo realizado no Mato Grosso, que a poluição do ar 42% e os agravos respiratórios 50% foram os problemas mais citados pela população das áreas de influência direta. Ainda neste estudo, foi verificado aumento das taxas de internações hospitalares por agravos respiratórios em crianças menores de 5 anos, com incremento de 39% quando associadas ao maior rendimento de cana-de-açúcar, e de 26% quando associadas às maiores concentrações atmosféricas de material particulado nos municípios analisados. Outros estudos ^19^^,^^20^^,^^21^, realizados em São Paulo, têm demonstrado os impactos no ambiente e na saúde, particularmente com comprometimento do sistema respiratório, decorrentes do processo de queimadas e emissão de particulados durante a produção de cana-de-açúcar no Brasil, com aumento de internações notadamente em menores de 5 anos.

O Estado de Pernambuco, localizado na Região Nordeste, destacou-se como o segundo maior produtor de açúcar e terceiro maior produtor de etanol das regiões Norte e Nordeste no ano de 2020. Neste mesmo período, a produção de etanol registrou um incremento de 22,9% a mais do que o ano anterior, sendo o 3º estado do país que mais ampliou sua produção no setor. Cerca de 99,3% da colheita realizada no estado ocorre a partir do corte manual e queima prévia da palha da cana-de-açúcar ^22^. Diante da necessidade de construir políticas públicas de proteção à saúde humana e ambiental nos territórios inseridos nessa cadeia produtiva, este estudo objetiva analisar a relação entre as internações hospitalares por doenças respiratórias e a queima regular da palha de cana-de-açúcar em Pernambuco.

## Método

Trata-se de um estudo ecológico de tendência temporal com abordagem descritiva e analítica ^23^, sistematizado em uma série histórica de 11 anos, correspondente ao período de 2008 a 2018. Observou-se a distribuição mensal das hospitalizações por causas selecionadas do capítulo X da Classificação Internacional de Doenças - 10ª revisão CID-10, registradas no Sistema de Informações Hospitalares do Sistema Único de Saúde SIH/SUS, são elas: pneumonia, asma, bronquite e bronquiolite aguda. Foram analisadas as faixas etárias de crianças menores de 5 anos e idosos maiores de 60 anos, por serem grupos mais vulneráveis a este conjunto de agravos.

**Figura 1:**
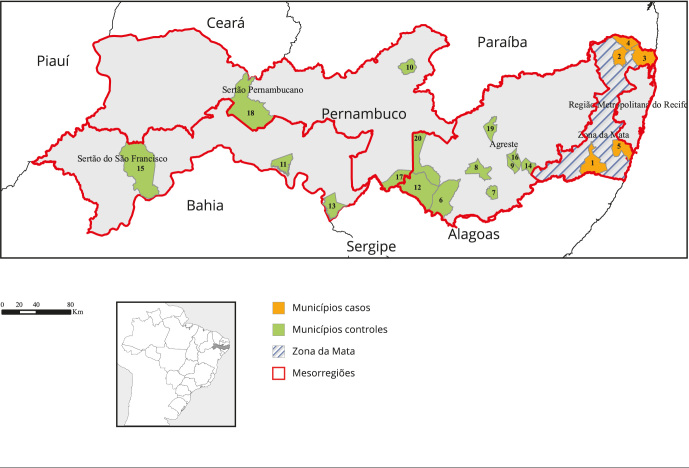
Localização geográfica dos municípios casos e controles, distribuídos por mesorregiões do Estado de Pernambuco, Brasil.

Foi realizado um comparativo das taxas de internação hospitalar entre os conjuntos de municípios casos e controles do Estado de Pernambuco. O critério utilizado para a definição dos cinco municípios casos foi a condição de serem os territórios de maior área plantada de cana-de-açúcar no estado: Água Preta, Aliança, Sirinhaém, Itambé e Goiana ^24^^,^^25^. O número de municípios controles foi definido considerando a proporção de um para três 1 caso: 3 controles, estabelecida para assegurar maior comparabilidade entre os grupos. Para a seleção dos municípios controles, foram adotados os seguintes critérios de inclusão: a não registrar nenhum hectare de área plantada de cana-de-açúcar entre 2008 e 2018; b não ser centro urbano desenvolvido ou pertencer à Região Metropolitana da capital, Recife; c não ter polo industrial desenvolvido; d não fazer fronteira com nenhum importante produtor de cana-de-açúcar; e e não ser município polo agrícola, gesseiro, cimenteiro ou de confecção. Foram excluídos os municípios que não se enquadraram em pelo menos um desses critérios. Após este procedimento amostral, dentre os 185 municípios pernambucanos, foram selecionados 15 como grupo controle: Águas Belas, Brejão, Caetés, Calçado, Ingazeira, Itaíba, Jatobá, Jurema, Lagoa Grande, Lajedo, Manari, Sanharó, Tupanatinga, Itacuruba e Salgueiro Figura 1.

Municípios casos: 1 Água Preta; 2 Aliança; 3 Goiana; 4 Itambé; e 5 Sirinhaém. Municípios controles: 6 Águas Belas; 7 Brejão; 8 Caetés; 9 Calçado; 10 Ingazeira; 11 Itacuruba; 12 Itaíba; 13 Jatobá; 14 Jurema; 15 Lagoa Grande; 16 Lajedo; 17 Manari; 18 Salgueiro; 19 Sanharó; e 20 Tupanatinga.

Para análise estatística das taxas de internações hospitalares referentes às doenças respiratórias foi utilizado o teste não paramétrico de Mann-Whitney. O método é recomendado para comparar amostras independentes e utiliza a mediana como medida que melhor representa o centro da distribuição ao longo da série histórica, auxiliando na observação dos valores e suas condições de igualdades ou diferenças estatísticas ^26^.

Para descrever uma possível relação existente entre o aumento das hospitalizações por doenças respiratórias e a ocorrência de queimadas da palha de cana-de-açúcar, foram cruzados os dados mensais coletados a partir do SIH/SUS e os dados dos focos de calor, obtidos na plataforma BDQUEIMADAS http://terrabrasilis.dpi.inpe.br/queimadas/bdqueimadas/ do Instituto Nacional de Pesquisas Espaciais INPE, sistema que compila registros de queimadas detectadas por um conjunto de satélites a partir de pontos de alta temperatura presentes na superfície terrestre. A identificação das áreas de maior densidade de calor sujeita-se à resolução espacial do sistema sensor de cada satélite, sendo a extensão mínima detectável correspondente a uma área de 30 metros de comprimento por 1 metro de largura de área queimada, segundo validação de campo. O comportamento sazonal de ambas as variáveis foi descrito a partir de gráficos de série histórica temporal e a análise da associação entre elas ocorreu pela correlação de Pearson, representada por gráficos de dispersão.

## Resultados

### Internações hospitalares por agravos respiratórios

Nos municípios casos, as taxas de hospitalizações por doenças respiratórias nos grupos etários de menores de 5 anos e maiores de 60 anos foram superiores quando comparadas às taxas dos municípios no grupo controle.

Ao comparar as taxas de internações hospitalares mensais nos municípios casos e controles, pode-se verificar, a partir do teste U de Mann-Whitney, que houve diferença estatística significante em ambos os grupos etários p < 0,005, média dos pontos de 106,39 para o grupo controle e 158,61 para o grupo de casos em crianças menores de 5 anos, sendo U = 5.266 e p < 0,005, média dos pontos de 101,92 para o grupo controle e 163,08 para o grupo de casos em idosos acima de 60 anos, sendo U = 4.676 Tabela 1.

**Tabela 1:** Análise estatística não paramétrica das taxas mensais de internações hospitalares por pneumonia, asma, bronquite e bronquiolite aguda e distribuição estratificada por percentis de suas medianas, dos municípios casos e controles. Pernambuco, Brasil, período de 2008 a 2018.

Grupo	N	Média dos pontos	Soma dos pontos	Teste U de Mann-Whitney	Z	Valor de p	Percentis		
							10	50	90
Crianças < 5 anos									
Controle	132	106,39	14.044,0	5.266,00	5,55	2.77^-8^	0,54	1,02	1,75
Caso	132	158,61	20.936,0				0,76	1,42	2,51
Idosos > 60 anos									
Controle	132	101,92	13.454,0	4.676,00	6,50	7,68^-11^	0,36	0,58	0,79
Caso	132	163,08	21.526,0	0,50	0,75	1,08

Considerando-se as taxas de hospitalizações, distribuídas mensalmente ao longo da série histórica analisada, observou-se que, em todos os percentis, a mediana dos municípios casos é superior à mediana dos municípios controles para ambas as faixas etárias de maior vulnerabilidade.

No percentil 50, para idosos acima de 60 anos, a razão das medianas aponta uma taxa 28% mais elevada nos municípios casos. Para crianças menores de 5 anos, a taxa mediana das hospitalizações por agravos respiratórios é 40% maior nos municípios casos.

### Sazonalidade das hospitalizações por agravos respiratórios

**Figura 2:**
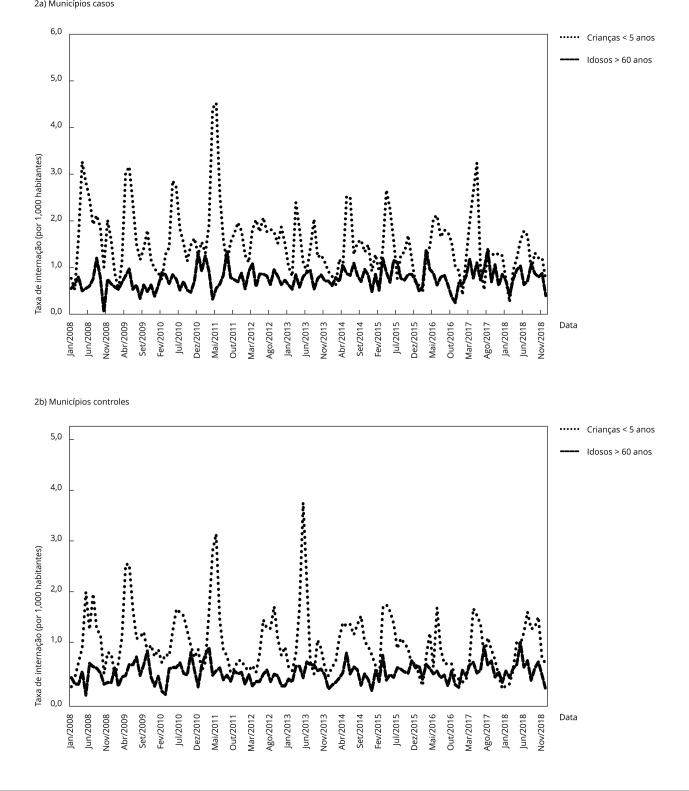
Distribuição mensal das taxas de internações hospitalares por pneumonia, asma, bronquite e bronquiolite aguda nos municípios casos e controles, estratificada em crianças menores de 5 anos e idosos maiores de 60 anos. Pernambuco, Brasil, período de 2008 a 2018.

O comportamento das taxas de internações hospitalares por agravos respiratórios em idosos e crianças menores de 5 anos, ao longo do curso da série histórica analisada, indica um padrão sazonal semelhante em municípios casos e controles. As hospitalizações por estes agravos apresentam-se, em geral, mais baixas nos meses de janeiro, sofrendo importante incremento no segundo trimestre, com registros mais elevados nos meses de abril a julho Figura 2.

Nos municípios casos, o mês de fevereiro marca o fim do período da queima da palha da cana-de-açúcar. Em abril registra-se o início das chuvas de outono/inverno na Zona da Mata pernambucana e o consequente aumento da umidade relativa do ar que, devido à proximidade com a costa litorânea, pode chegar em torno de 90% nos períodos mais chuvosos dos municípios da região. Excepcionalmente, no terceiro trimestre do ano 2011, houve um incremento discreto nas hospitalizações para a faixa etária acima de 60 anos nos municípios casos Figura 2.

A comparação das taxas de internações hospitalares em ambos os grupos etários evidencia mais casos de hospitalização por este grupo de agravos entre crianças menores de 5 anos em todos os meses do ano.

### Ocorrência de focos de calor e hospitalizações por agravos respiratórios

Observou-se que a ocorrência de focos de calor nos municípios que cultivam a cana-de-açúcar é superior aos não produtores Tabela 2.

**Tabela 2:** Distribuição das médias mensais das ocorrências de focos de calor nos municípios casos e controles. Pernambuco, Brasil, período de 2008 a 2018.

Mesorregiões/Municípios	Janeiro	Fevereiro	Março	Abril	Maio	Junho	Julho	Agosto	Setembro	Outubro	Novembro	Dezembro
Zona da Mata casos												
Água Preta	12,55	9,09	4,73	5,09	0,73	0,36	0,18	0,18	1,91	6,64	14,09	17,45
Aliança	15,00	5,18	3,18	0,82	0,55	0,73	0,45	1,55	8,00	20,00	30,91	21,73
Goiana	17,91	7,27	0,82	1,55	0,27	0,09	0,64	3,00	21,00	35,35	42,64	37,91
Itambé	16,27	9,36	4,55	0,55	0,45	0,36	0,09	4,09	7,64	14,91	24,82	29,00
Sirinhaém	11,73	10,18	3,91	1,55	0,45	0,64	0,45	0,27	3,64	10,82	12,27	11,55
Agreste controle												
Águas Belas	5,36	7,55	9,45	5,82	2,45	1,45	0,18	0,55	1,27	3,09	6,27	5,09
Brejão	0,18	0,09	0,45	0,09	0,00	0,00	0,00	0,00	0,00	0,00	0,09	0,18
Caetés	0,18	0,00	0,00	0,00	0,09	0,00	0,00	0,00	0,18	0,27	0,38	0,73
Calçado	0,18	0,18	0,18	0,45	0,09	0,00	0,09	0,00	0,00	0,45	0,18	0,55
Itaíba	6,09	7,00	4,55	3,64	1,91	1,18	0,36	0,91	1,27	2,64	7,45	8,73
Jurema	0,36	0,09	0,91	0,27	0,09	0,00	0,45	0,09	0,18	0,18	0,09	0,00
Lajedo	0,18	0,36	0,36	0,36	0,00	0,00	0,18	0,09	0,18	0,18	0,09	0,00
Sanharó	6,27	4,27	2,82	3,00	1,82	0,73	0,18	0,09	0,73	1,91	3,09	5,64
Tupanatinga	8,00	6,36	6,64	3,45	2,82	1,00	0,64	1,18	1,91	5,09	5,82	8,55
Sertão Pernambucano controle												
Ingazeira	4,36	2,73	0,73	0,64	0,09	0,00	0,55	1,18	1,09	3,73	8,91	10,91
Manari	2,09	1,73	1,64	1,09	0,36	0,09	0,00	0,45	0,00	1,18	1,82	2,55
Salgueiro	5,64	2,00	1,27	0,82	0,82	0,82	1,27	3,00	5,91	14,09	21,45	16,27
Sertão do São Francisco controle												
Itacuruba	1,27	1,27	0,91	0,45	0,27	0,55	0,64	0,09	0,18	1,09	1,27	1,00
Jatobá	1,18	0,27	0,00	0,09	0,36	0,00	0,00	0,36	0,36	0,18	0,18	0,45
Lagoa Grande	2,91	1,91	2,36	1,55	1,27	1,55	1,36	3,18	9,64	13,64	8,82	3,45

Entretanto, a análise da distribuição temporal das ocorrências dos focos de calor, em associação com as taxas de hospitalizações mensais por agravos respiratórios em ambas as faixas etárias, não apresentou correlação estatística significativa de acordo com os parâmetros obtidos pelo modelo de análise utilizado. Diferente do padrão sazonal das hospitalizações por agravos respiratórios, mais prevalentes entre os meses de abril a julho, a distribuição mensal dos focos de calor nos municípios canavieiros concentra-se entre os meses de agosto e fevereiro Tabela 2, período equivalente à colheita dos monocultivos de cana-de-açúcar produzido nos municípios casos.

**Figura 3:**
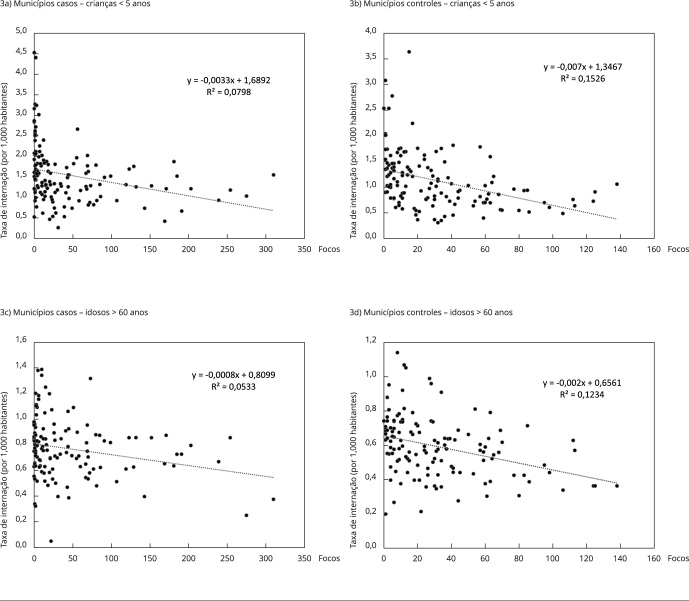
Dispersão da correlação das taxas mensais de internação por agravos respiratórios em ambas as faixas etárias e ocorrência de focos de calor nos municípios casos e controles. Pernambuco, Brasil, período de 2008 a 2018.

Em ambos os grupos de municípios analisados e para ambas as faixas etárias mais vulneráveis, a tendência é, inclusive, de leve queda nas taxas de internação à medida que se aumentam os valores de focos de calor, embora estas sofram incremento significativo nos três meses subsequentes ao início do período de queima Figura 3.

## Discussão

### Sazonalidade das hospitalizações por agravos respiratórios

Associações significativas entre a ocorrência de hospitalizações por doenças respiratórias e a poluição do ar têm sido observadas em diversos estudos ^27^^,^^28^^,^^29^^,^^30^. Em relação aos danos à saúde decorrentes da exposição às emissões de queimadas, estudos recentes evidenciam tanto aumento no número de internações por doenças respiratórias ^29^^,^^31^^,^^32^, quanto por outras doenças, em especial cardiovasculares ^29^^,^^33^, bem como incremento no número de óbitos ^31^^,^^33^^,^^34^.

O aumento na taxa de internação hospitalar por condições respiratórias em crianças menores de 5 anos tem sido associado à queima de biomassa da cana-de-açúcar em estudos desenvolvidos em regiões produtoras no Brasil ^18^^,^^19^^,^^20^^,^^21^^,^^35^. Evidencia-se aumento de até 38% nas internações oriundas da exposição crônica ^19^, um risco relativo de 15% a mais de internação dependendo da variação do material particulado emitido ^34^. Não obstante, Mauro et al. ^35^ sugerem que os desfechos respiratórios nas crianças podem ser inversamente proporcionais à idade, carecendo de maiores análises.

Em outros cenários, a exemplo das poluições emitidas na Região Amazônica por outras fontes de emissão de particulados de queimadas, Requia et al. ^29^ registraram aumento de 21% nas admissões hospitalares por agravos respiratórios entre crianças com idade ≤ 5 anos, e de 19% para pessoas com idade ≥ 65 anos. Ignotti et al. ^17^ também observaram associações semelhantes, encontrando aumento de 8% na hospitalização de crianças e 10% na de idosos.

No entanto, ainda que as condições socioambientais de exposição sejam as variantes centrais para o surgimento das doenças aqui analisadas, as condições fisiológicas e patológicas típicas de cada grupo etário podem constituir e distinguir suscetibilidades específicas ao adoecimento ^36^.

Por outro lado, a sobreposição de variantes climáticas com os impactos da poluição atmosférica proveniente da queima de biomassa é um fenômeno influente nas taxas de hospitalizações por agravos respiratórios em grupos etários de maior vulnerabilidade. Nota-se um caráter sazonal para os desfechos respiratórios mais sensíveis às condições ambientais e sociais ^9^^,^^17^^,^^37^^,^^38^. As análises estratificadas por idade apontam associação entre o material particulado fino presente em emissões atmosféricas, decorrentes de queimadas, e incremento na ocorrência de asma, bronquite e doenças respiratórias em geral em crianças ^39^. Nos idosos, destacam-se a doença pulmonar obstrutiva crônica DPOC, infecções e agravos respiratórios em geral ^34^^,^^40^.

Chama atenção que, embora o incremento das taxas de hospitalizações analisadas neste estudo não coincidam com o ciclo de queima da cana-de-açúcar, elas convergem para o período de chuvas no Nordeste brasileiro. Conforme aponta Gomes et al. ^41^, as fortes chuvas e elevadas taxas de umidade relativa do ar marcam o outono/inverno desta região e predispõem à maior proliferação de fungos e mofo em ambientes fechados. Some-se a isto, variantes individuais de comportamento como a permanência e aglomeração em lugares com baixa ventilação, cuja saturação do ar torna-se ainda mais elevada quando há concentração de poluentes atmosféricos, sendo estes condicionantes relevantes para a sazonalidade das internações hospitalares por agravos respiratórios. De acordo com Sales ^42^, as variáveis de maior peso hierárquico na influência dos comportamentos climáticos sobre a ocorrência de agravos respiratórios são a concentração de material particulado MP_10_ e CO em suspensão atmosférica, seguida da umidade relativa e densidade demográfica.

Ampliando as análises anteriores, outros autores têm relacionado os baixos índices de umidade com o adoecimento respiratório nas regiões mais secas do país. Silva et al. ^6^ observaram que a média diária de material particulado fino MP_2,5_ foi 50% maior nos períodos de seca, quando havia diminuição da umidade relativa do ar e aumento da temperatura ambiente. Ainda verificaram o aumento de 10mg/m^3^ nos níveis de exposição ao MP_2,5_ durante todo o ano, sendo associado ao incremento de 12,1% nas médias móveis de hospitalizações de crianças por doenças respiratórias e 22% nos períodos de estação seca. Vasconcellos et al. ^43^ apontaram semelhanças na composição atmosférica entre uma região produtora de cana-de-açúcar, na época de queima de biomassa no interior do Estado de São Paulo, e o ar coletado no centro urbano da megacidade durante a estação seca.

Vasconcelos et al. ^44^ destacaram a influência dos eventos de precipitação durante a primavera e verão como fatores importantes para a remoção desse material em suspensão na atmosfera.

### Ocorrência de focos de calor e hospitalizações por agravos respiratórios

A análise da distribuição das ocorrências mensais de focos de calor neste estudo expôs um padrão de regularidade e de maiores frequências nos municípios produtores de cana-de-açúcar ao longo da série histórica, conforme observado em outros estudos ^8^^,^^10^^,^^11^^,^^37^, embora mostre-se divergente do padrão sazonal das hospitalizações por agravos respiratórios nos municípios casos e controles, confluindo com outros trabalhos ^31^^,^^37^^,^^45^.

Entretanto, no estudo de Paraiso & Gouveia ^20^ observou-se uma relação entre o aumento dos focos de calor da queima de biomassa p = 0,008 e as internações de menores de 5 anos. A associação entre a prática regular de queimada e consequências negativas para saúde respiratória também foi evidenciada por Castro et al. ^46^ e Souza ^47^, quando verificada uma correlação positiva entre a variação das ocorrências de focos de calor e registros de agravos respiratórios em menores de 4 e 5 anos, em seus respectivos estudos. Mais recentemente, Ramos et al. ^48^ encontraram uma diferença significativa do número de internações por doenças respiratórias no período da queima, em comparação ao período de não queima p = 0,011. Castro et al. ^34^ evidenciaram correlações positivas entre focos de calor provenientes de queimadas com taxas de mortalidade em idosos por doenças respiratórias agudas p = 0,0001 para faixa etária de 65 a 74 anos e p = 0,0007 para idosos com 74 anos e mais e por DPOC em idosos p = 0,0003 para faixa etária de 65 a 74 anos e p = 0,0001 para idosos com 74 anos e mais.

Salienta-se que os desfechos na saúde respiratória podem não ocorrer de forma instantânea e simultânea ao evento das queimadas, variando seu surgimento ao longo do tempo e, conforme reportam Requia et al. ^29^, pode haver um aumento de até 20% após cinco dias do registro da queimada.

A partir dos resultados compartilhados em nosso trabalho, levanta-se a hipótese de que a exposição persistente e sazonal, em territórios com maiores ocorrências de focos de calor, pode produzir hipersensibilidade populacional aos particulados tóxicos e maior predisposição aos agravos respiratórios, como observado nas taxas elevadas de hospitalização nos municípios casos ao longo de todos os meses do ano, no período analisado. Existem discussões acerca da plausibilidade biológica desta ocorrência, ancoradas em evidências anunciadas por estudos antecedentes, como o de Goto et al. ^49^, que observou os efeitos da queima da biomassa na depuração mucociliar em trabalhadores cortadores de cana-de-açúcar, indicando um aumento da susceptibilidade dos trabalhadores às doenças respiratórias associadas a esta prática agrícola. Outro estudo congênere verificou o caráter central das variáveis de tempo e espaço na determinação dos níveis de maior exposição aos focos de calor, sob os quais estão submetidos aproximadamente 50% dos moradores das áreas adjacentes que relataram manifestações de sintomas de asma durante os 12 meses do ano ^11^.

Mnatzaganian et al. ^50^ apontam uma possível relação direta entre a extensão da área queimada e maiores prevalências por agravos agudos do aparelho respiratório. Sabe-se também que a exposição humana à fuligem tóxica, proveniente da queima da biomassa, é maior nas localidades encontradas às proximidades dos focos de calor ^11^.

Para além da sintomatologia clínica dos agravos respiratórios, a exposição ao material particulado em suspensão atmosférica incide diretamente sobre mecanismos bioquímicos, eventos intra, extra e intercelulares, estendendo suas consequências para outros sistemas do corpo humano, a exemplo do homeostático, sanguíneo e imune. Inclusive, seu potencial mutagênico precisa ser levado em consideração quando a exposição persistente abrange dimensões populacionais ^29^.

Como visto, as elevadas taxas de hospitalizações por agravos respiratórios nos grupos populacionais aqui estudados, nos municípios produtores de cana-de-açúcar, sugerem possível associação com a exposição crônica aos particulados tóxicos, emitidos pela queima regular de biomassa. Endossa a necessidade de revisão e reestruturação das práticas seculares, e ainda operantes, do monocultivo extrativista da cana-de-açúcar, bem como demanda a construção de políticas públicas de proteção à saúde humana e ambiental nos territórios inseridos na cadeia produtiva sucroenergética.

Conforme Oliveira & Anunciação ^51^ apontam, existe uma vulnerabilidade ecossistêmica associada à prática regular de queima da biomassa, evidenciada pela distorção no ciclo de chuvas. Segundo os autores, uma vez que há relação entre a ocorrência de focos de calor e o baixo índice de precipitação, o impacto climático nos territórios produtivos que conservam a prática regular da queima de biomassa pode ser observado.

É urgente a estruturação de uma vigilância em saúde de base territorial nos municípios submetidos às queimadas regulares. Certamente, o monitoramento ambiental e em saúde deve ser atrelado à oferta de uma rede de assistência adequada e especializada com fluxos de referência e contrarreferência bem estabelecidos entre os serviços, assegurando a continuidade do cuidado integrado em saúde. Ressalta-se a necessidade de outras análises futuras preocupadas com a elucidação da hipótese que associa as altas taxas de hospitalizações aos períodos de maiores concentrações atmosféricas dos particulados emitidos pela queima da palha da cana-de-açúcar.

Dados os limites relacionados à natureza dos dados secundários analisados neste artigo, não foi possível espacializar a influência dos focos de calor sobre os agravos respiratórios que acometem a população exposta nos territórios em análise. Por um lado, as limitações que envolvem o uso dos dados de focos de calor variam desde restrições na detecção de ocorrência e delimitação da extensão da área queimada, até falhas devido às influências de variações naturais ^47^. Por outro, os dados registrados no SIH/SUS referem-se apenas aos casos graves que necessitaram de hospitalização no período analisado, excluindo os casos leves e moderados assistidos ou não por unidades da atenção primária, serviços ambulatoriais e urgências. Além disso, a menor unidade de análise espacial disponível no SIH/SUS é o município, inviabilizando uma análise mais precisa da vulnerabilidade das populações que residem nas proximidades das áreas submetidas às queimadas. Como os municípios são territorialmente pequenos, a espacialização dos casos, considerando a ampla e disseminada distribuição dos focos de calor ao longo dos anos, conjectura-se que não haveria diferenças significativas quanto aos níveis de exposição dos casos.

A despeito da importância da realização de estudos que evidenciem a poluição atmosférica pela queima de biomassa e sua relação com doenças respiratórias, em particular da cana-de-açúcar, dada sua importância na economia brasileira, observou-se uma escassez de pesquisas no Estado de Pernambuco e na Região Nordeste do país. Como a taxa de mecanização da colheita da cana-de-açúcar é de apenas 22,9% nas regiões Norte e Nordeste, as queimadas e a colheita manual ainda se mantêm nesses territórios ^22^. Destaca-se que, para a safra 2022-2023, Pernambuco foi o estado que apresentou o maior aumento da área em produção no país, com ganho de 10,9 mil hectares ^2^, reforçando a importância da realização de estudos dessa natureza na região.

## Conclusão

Os achados desta pesquisa sugerem possível associação de problemas respiratórios com a exposição crônica aos particulados emitidos pela queima de biomassa, comprometendo a saúde de grupos vulneráveis nos territórios do agronegócio em Pernambuco. Não obstante, endossam a necessidade de substituição das queimadas no monocultivo da cana-de-açúcar, bem como a estruturação de políticas públicas de proteção à saúde humana e ambiental.
